# Improvement of the banana “*Musa acuminata*” reference sequence using NGS data and semi-automated bioinformatics methods

**DOI:** 10.1186/s12864-016-2579-4

**Published:** 2016-03-16

**Authors:** Guillaume Martin, Franc-Christophe Baurens, Gaëtan Droc, Mathieu Rouard, Alberto Cenci, Andrzej Kilian, Alex Hastie, Jaroslav Doležel, Jean-Marc Aury, Adriana Alberti, Françoise Carreel, Angélique D’Hont

**Affiliations:** CIRAD (Centre de coopération Internationale en Recherche Agronomique pour le Développement), UMR AGAP, TA A-108/03, Avenue Agropolis, F-34398, Montpellier, cedex 5 France; Bioversity International, Parc Scientifique Agropolis II, 34397, Montpellier, Cedex 5 France; Diversity Arrays Technology, Yarralumla, Australian Capital Territory 2600 Australia; BioNano Genomics, 9640 Towne Centre Drive, San Diego, CA 92121 USA; Institute of Experimental Botany, Centre of the Region Hana for Biotechnological and Agricultural Research, Šlechtitelů 31, CZ-78371 Olomouc, Czech Republic; Commissariat à l’Energie Atomique (CEA), Institut de Genomique (IG), Genoscope, 2 rue Gaston Cremieux, BP5706, 91057 Evry, France

**Keywords:** *Musa acuminata*, Genome assembly, Bioinformatics tool, Paired-end sequences, GBS, Genome map

## Abstract

**Background:**

Recent advances in genomics indicate functional significance of a majority of genome sequences and their long range interactions. As a detailed examination of genome organization and function requires very high quality genome sequence, the objective of this study was to improve reference genome assembly of banana (*Musa acuminata*).

**Results:**

We have developed a modular bioinformatics pipeline to improve genome sequence assemblies, which can handle various types of data. The pipeline comprises several semi-automated tools. However, unlike classical automated tools that are based on global parameters, the semi-automated tools proposed an expert mode for a user who can decide on suggested improvements through local compromises. The pipeline was used to improve the draft genome sequence of *Musa acuminata.* Genotyping by sequencing (GBS) of a segregating population and paired-end sequencing were used to detect and correct scaffold misassemblies. Long insert size paired-end reads identified scaffold junctions and fusions missed by automated assembly methods. GBS markers were used to anchor scaffolds to pseudo-molecules with a new bioinformatics approach that avoids the tedious step of marker ordering during genetic map construction. Furthermore, a genome map was constructed and used to assemble scaffolds into super scaffolds. Finally, a consensus gene annotation was projected on the new assembly from two pre-existing annotations. This approach reduced the total *Musa* scaffold number from 7513 to 1532 (i.e. by 80 %), with an N50 that increased from 1.3 Mb (65 scaffolds) to 3.0 Mb (26 scaffolds). 89.5 % of the assembly was anchored to the 11 *Musa* chromosomes compared to the previous 70 %. Unknown sites (N) were reduced from 17.3 to 10.0 %.

**Conclusion:**

The release of the *Musa acuminata* reference genome version 2 provides a platform for detailed analysis of banana genome variation, function and evolution. Bioinformatics tools developed in this work can be used to improve genome sequence assemblies in other species.

**Electronic supplementary material:**

The online version of this article (doi:10.1186/s12864-016-2579-4) contains supplementary material, which is available to authorized users.

## Background

The first two plant genomes to be sequenced were *Arabidopsis* and rice. Their sequences were obtained by sequencing a minimum tiling path of bacterial artificial chromosome (BAC) clones selected from physical maps. Since then, the number of sequenced plant genomes has increased steadily each year, thanks to considerable decrease in costs and increase in throughput of sequencing technologies [1–3]. Nowadays, most genome assemblies are produced after whole genome shotgun sequencing (WGS) using Next Generation Sequencing (NGS). WGS is based on three main steps: i) assembling raw sequence reads into larger sequences called contigs; ii) building bridges between contigs using end-sequenced DNA fragments of various lengths (*e.g* BACs, fosmids, plasmids, large insert size libraries) to generate scaffolds; iii) anchoring scaffolds to chromosomes using genetic mapping data to produce pseudo-molecules.

A major challenge is to generate highly contiguous sequence assemblies from short reads in genomes characterized by sequence redundancy, which is a typical situation for plants. The main source of redundancy is transposable elements (TE) that represent a large part of plant genomes (from 14 % in *Arabidopsis* to 80 % in wheat) (reviewed in [4]). Another source of difficulties are paralogous genes [5] resulting from various types of duplications processes including whole genome duplication (WGD) that occurred frequently during the evolution of plants [6] or segmental duplication of various sizes. Repeated sequences are often assembled into a single collapsed region during the assembly steps [7]. Once created, a collapsed region is linked to multiple other genomic regions leading to conflicts. Automatic assemblers then face two problematic options, either to assemble anyway with a risk to misassemble non-contiguous regions or to prematurely stop the sequence assembly process. These constraints are exacerbated with short insert-size paired reads since the insert size will not span repeat elements. Conversely, scaffolding with only very large insert size libraries (i.e. BAC-end sequences) limits the integration of small scaffolds in the final assembly.

New approaches are continuously being developed to improve genome sequence assemblies. They include longer read sequencing, high coverage medium and large insert size libraries [8, 9], optical maps [10–12], which improve contigs assembly into scaffolds, and genotyping by sequencing (GBS), which has been used to assemble scaffolds into pseudo-molecules [13, 14]. In contrast to tremendous advances in high-throughput sequencing, assembling sequences remains a substantial endeavor [15]. Several automated programs have been developed to improve draft genome sequence assemblies such as Bambus [16], SOPRA [17], MIP [18], SSPACE [19], Opera [20], GRASS [21], SCARPA [22], SSPACE-LongRead [23], SOAP-de-novo2 [24], GapFiller [25] and PAGIT [26]. However, these programs were designed for assembling contigs into scaffolds and/or filling unknown regions, and are running under a compromise between the quantity and quality of the assembly. This compromise results in a significant proportion of misassembled, un-scaffolded and un-filled regions.

A draft genome sequence assembly of banana (*Musa acuminata*, 2n = 22, 1C = 523 Mbp)*,* was produced recently using the WGS strategy [27]. The sequence was obtained from a doubled-haploid plant of cv. Pahang and represented a major step forward in understanding the structure and evolution of the banana genome [27, 28]. Specific ancestral whole genome duplications were identified within the *Musa* lineage and their impact on gene fractionation and expression patterns was characterized [29]. Being the first monocotyledon genome sequence outside the Poales, the sequence provided an essential bridge for comparative genome analysis in plants e.g. [27, 28, 30–34].

According to criteria outlined by [35], this genome sequence can be classified as high quality draft. However, there has been an obvious room for improvement, including the reduction of the number of scaffolds (7573) and the number of scaffolds not anchored to one of the eleven chromosomes (30 % of the draft assembly). Here we describe a significant improvement of the first *Musa acuminata* draft reference genome sequence and the bioinformatics tools that we developed and used in this work. The work comprised: i) detection and correction of sequence misassemblies, ii) merging scaffolds, and iii) integration of many previously un-anchored scaffolds to the 11 pseudo-molecules. In addition, conciliation between existing genome annotations was made.

## Methods

### Sequence data

The first draft reference sequence of banana (*Musa acuminata*) [27] was produced from DNA of a doubled-haploid plant of cv. ‘Pahang’ (DH-Pahang) using reads obtained by 454 sequencing (ERX166948 to ERX167027), Sanger 10 kb fosmid paired-reads (available on the Banana Genome Hub, http://banana-genome.cirad.fr/download), Sanger BAC-end reads (available on the Banana Genome Hub, http://banana-genome.cirad.fr/download) and 330 bp pair-end illumina sequences (ERX179491 to ERX179503). In the present work a 5 kb mate-pair library of DH-Pahang was created and sequenced using illumina HiSeq 2000 to 40x genome coverage. The reads obtained were trimmed and filtered following three criteria: (1) trimming of both read ends until base quality is higher or equal to 20; (2) read trimming at the second unknown base in the sequence; and (3) read larger or equal to 30 bases were conserved.

### Single molecule mapping

Genome map of DH-Pahang genome was constructed using BioNano Irys System (BioNano Genomics, San Diego, USA). High molecular weight (HMW) DNA was prepared according to [36]. Briefly, a liquid suspension of intact cell nuclei was prepared by mechanical homogenization of formaldehyde-fixed tissues of unopened (cigar) leaves. The nuclei in the homogenate were stained by DAPI (4′,6-diamidino-2′-phenylindole), the nuclei in G_1_ phase of cell cycle were purified by flow cytometric sorting and embedded in agarose miniplugs. HMW DNA was then purified and labeled using IrysPrep Reagent Kit (BioNano Genomics). The labelling was done with fluorescent nucleotide analogs at all Nt.BspQI nicking endonuclease sites. Single molecules were linearized in nanochannel arrays, imaged. A total of 426,846 molecules, with a N50 of 153 kb, representing a cumulated length of 65,719 Mb with an average label density of 9.4 labels/100 kb were generated and *de novo* assembled using a layout-overlap-consensus method. The *de novo* map assembly yielded 464 Mb with a map N50 of 715 kb.

### Genetic markers

A total of 180 individuals among the 268 individuals of a self-progeny of the ‘Pahang’ accession (PT-BA-00267) obtained at the CIRAD research station in Guadeloupe were genotyped using the DArTseq technology [37]. A total of 9,968 co-dominant (SNP) and 16,233 dominant markers were generated using a *Pst*I-*Mse*I enzyme combination. These markers were used in addition to the 768 SSR and 497 DArT markers previously used to anchor the *Musa acuminata* genome assembly. Out of the 268 individuals in the mapping population, 91 individuals were genotyped with all types of markers, 178 individuals with both DArT and DArTseq markers, 91 individuals with both DArTseq and SSR markers and 176 individuals with both DArT and SSR markers. The markers were filtered independently for each marker type on the basis of the following criteria: no more than 20 % missing data, no less than 10 % heterozygous or dominant and no less than 1.5 % homozygous for at least one homozygous state, resulting in 23,430 markers. The choice of these relatively non-stringent parameters was motivated by large segregation distortions that were previously observed in chromosome 1 and chromosome 4 in the segregating population [27].

### Gene annotation

Two gene annotations of the *Musa acuminata* draft genome sequence were available for the initial assembly. The first corresponded to the annotation published by [27], in addition to approximately 1000 genes curated by human expertise before 08 December 2014 (http://banana-genome.cirad.fr/). The second one was the NCBI RefSeq genome annotation released the 7 October 2014 (ftp://ftp.ncbi.nlm.nih.gov/genomes/refseq/plant/Musa_acuminata/) and generated with the NCBI Eukaryotic Genome Annotation Pipeline.

### Bioinformatics pipeline

**Fig. 1:**
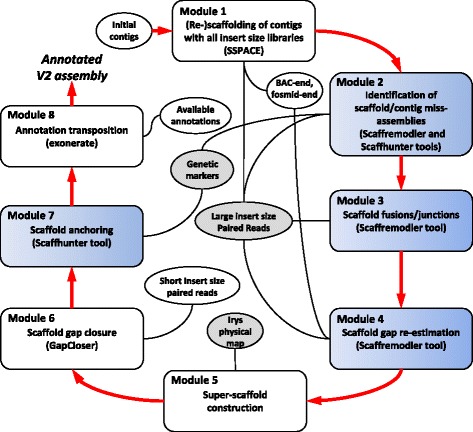
Overview of the pipeline used to improve the *Musa* draft genome sequence. Ellipses correspond to input data and *grey ellipses* indicate new data acquired for the improvement of the assembly. *Boxes* corresponds to bioinformatics tools, the ones in *blue* are new and made available through *Scaffremodler* and *Scaffhunte*r toolboxes respectively (see Additional file 1)

An overview of the pipeline used to improve the banana draft genome assembly is shown in Fig. 1. It is divided into 8 distinct modules corresponding to different and optional operations. This pipeline exploited several tools that we have developed and which are available under Scaffhunter and Scaffremodler toolboxes. The first one exploits genetic mapping data and the second one Large insert size Paired Reads (LPR). They are described in details in the Additional file 1.

#### Module 1: (Re-)scaffolding of contigs

This module used *SSPACE* [23] and exploited large insert size paired reads (LPR) to perform a new scaffolding of the existing contigs. The scaffolding process was divided into as many steps as the number of sequenced libraries with distinct inserts sizes. The libraries were used by increasing insert size order; scaffolding parameters were optimized for each step. To prevent accumulation of scaffolding errors, the first library was used with more stringent parameters (-a 0.5, -k 20) than the second and third ones (-a 0.7, -k 1). For Sanger sequence libraries (i.e. BAC-end and fosmids-end sequences) reads were mapped as single end-reads using *BWA* [38]. Single location reads were used to reconstruct read-pairs that were stored in a tabulated file used by *SSPACE*.

#### Module 2: identification and splitting of scaffold/contig misassemblies

This module identified and split misassembled contigs/scaffolds using a combination of GBS genetic mapping data and LPR data. Genetic markers were grouped into linkage groups using JoinMap4.1 software [39]. No marker ordering was performed at this stage. In parallel, marker sequences were aligned to scaffolds using a consensus of *BWA*, *bowtie2* [40] and *BLAST* [41] and only single hits markers were conserved. Scaffolds harboring markers attributed to more than one linkage group were identified. LPR aligning (using bowtie2 in --very-sensitive mode) in these scaffolds were inspected to precisely locate the misassembly boundaries. The misassembled boundaries were identified based on the absence of overlap of read-pairs in the area and an increased proportion of discordant reads. Misassembled scaffolds were then split. The complete process and tools used for this module are described in Additional file 1.

#### Module 3: scaffold fusions/junctions

This module used LPR to identify scaffolds that should be inserted into larger ones (hereafter referred to as fusion) and scaffolds that should be end-joined (hereafter referred to as junction). LPR were aligned to the scaffolds using bowtie2 in --very-sensitive mode. Only single hit LPR were conserved. Redundant LPR were filtered out using MarkDuplicates tool of Picard (http://broadinstitute.github.io/picard/). Filtered LPR were then used to identify discordant read clusters, which were used to identify potential scaffold fusions and scaffold junctions. Potential scaffold fusions and junctions were then manually validated by inspecting circos [42] picture showing paired reads position in these regions. Fusion and junction performed were validated by aligning LPR along the corrected scaffolds using bowtie2 (in --very-sensitive mode) and mapped reads were inspected to ensure that the newly created junctions are spanned with reads mapped in the correct orientation. The complete process and tools used for this module are described in Additional file 1.

#### Module 4: scaffold gap re-estimation

In this module, the size of all remaining gaps (region composed of N) were re-estimated using all paired-reads (i.e. LPR, BAC-ends sequences and fosmid paired reads). Paired reads were aligned against scaffolds using bowtie2 in --very-sensitive mode for illumina reads and BWA with *mem* algorithm for Sanger reads. For each paired read library, gaps were re-estimated so that correctly orientated paired read overlapping a gap have an insert size corresponding to the expected median insert size of the library. For the 5 kb mate-pair library (illumina), at least 30 pairs were required to re-estimate a gap while for the 10 kb and BAC-end Sanger reads at least 2 and 1 pairs were required respectively. The complete process and tools used for this module are described in Additional file 1.

#### Module 5: super scaffold construction

This module exploits genome map to arrange scaffolds into super scaffolds. First, the sequence assembly fasta file was converted into the BioNano Irys map format by running an “in silico digest with the Nt.BspQI nicking endonuclease” of the sequence assembly using Knickers (http://www.bnxinstall.com/knickers/Knickers.htm). Only scaffolds larger than 20 kb with more than five sites were used, representing 613 scaffolds for a cumulative size of 437 Mb. Then, using BioNano’s proprietary alignment tool RefAligner [43, 44], the sequence maps were compared with Irys genome maps to find their best alignments; here only sequence maps with more than 5 labels (i.e. Nt.BspQI nicking endonuclease site) were used for comparison. The sequence-Irys map pairs with significant discordance were flagged and removed, with discordance defined as more than 5 consecutive labels not unaligned on both the sequence map and the Irys map. These pairs may represent chimeric assemblies due to sequencing errors or allelic differences. Then, the filtered sequence maps and filtered Irys maps were merged with RefAligner using a *p*-value of 10^−10^ based on [45] to create super scaffolds. This merging process was iterative, and the merge order was based on map similarity. The iterations stopped when all possible pairs were merged. A tabulated file locating scaffold sequence into the merged maps was then used to group scaffolds into super scaffolds. Original scaffolds were separated by Ns corresponding to their expected distance in the physical map.

#### Module 6: scaffold gap closure

This module exploited paired short insert size reads (330 b pair-end illumina) to close gaps in scaffold using GapCloser v1.12 program [24]. At the end of this module, all scaffolds were renamed according to their length.

#### Module 7: scaffold anchoring

This module used genetic markers obtained from a genetic mapping population to group, order and assemble scaffolds into pseudo-molecules. Our approach avoided the step of genetic map construction and a subsequent conciliation between genetic map and scaffolds. We used blocks of already ordered markers based on their position on scaffolds and first ordered them relative to each other, using UPGMA-like based approach. Then this first order was improved with permutation testing. The process can be decomposed into 4 steps:

1. Marker location on scaffolds using a consensus of *BWA*, *bowtie2* and *BLAST,*
2. Pairwise linkage LOD calculation between markers using JoinMap4.1,
3. Calculation of a first order using an UPGMA like approach on mean pairwise linkage LOD calculated between scaffolds,
4. Scaffold ordering and orientation optimization by performing scaffold permutations and re-orientations leading to maximization of a score calculated as follows:

$$ \mathrm{score}={\displaystyle \sum_{i=1,j=1,{x}_i<{x}_j}^n\left(1\hbox{-} \frac{\left({x}_j\mathit{\hbox{-}}{x}_i\right)}{\mathrm{n}}\right)\mathrm{L}\mathrm{O}{\mathrm{D}}_{\mathrm{ij}}} $$

with n the number of markers in the LG to order, x_i_ and x_j_ are the position of markers i and j in the tested order, and LOD_ij_ the LOD score between markers i and j. To optimize computation time and as order is not tested within scaffolds, i and j are markers from different scaffolds. Scaffold sequences were then assembled into pseudo-molecules. In addition to a fasta file containing ordered scaffold sequences separated by 100 N, an AGP file locating scaffolds into pseudo-molecules was generated. The complete process and tools used for this module are described in Additional file 1.

#### Module 8: annotation transposition

This module consisted of transposing annotations from the first draft genome sequence to the new assembly. Gene annotations (consisting in fasta putative transcripts) were transferred to the new assembly using Exonerate software [46] with the cdna2genome model and a maximum allowed intron size of 30 kb. Exonerate performed genomic searches and spliced alignments in a single run. Using a custom Perl script, based on the exonerate output, we transferred the annotation on a new GFF3 files, and generated a file of sequence identifier equivalence between the two releases. The script performed some quality checks by comparing protein-coding sequences before and after the transfer as some discrepancies may occur. In such case, the script used Blastp to align genes exons by exons. Since two annotations were available (the annotation performed by [27] and the one performed by NCBI) both annotations were transposed. An additional consensus annotation was generated using a custom script that selected between the two annotations version genes spanning the same genomic coordinates based on tags enclosed in the GFF3 files using the intersect function of BEDTools [47].

## Results

The original banana, *Musa acuminata*, draft reference genome assembly [27] was improved using the approach, tools and datasets as summarized in Fig. 1. The improvement was made in 8 successive steps.

### Contig scaffolding

The original 24,425 contigs published in the first version of the *Musa acuminata* reference genome [27] were re-assembled into scaffolds exploiting paired end data, which were used for original version of the assembly (Sanger 10 kb fosmid paired-reads, Sanger BAC-end reads), and new 5 kb mate-pair illumina sequences (40x coverage). Contigs were assembled into 2,267 scaffolds for a cumulated size of 439 Mb representing 84 % of the estimated size (523 Mb) of the DH-Pahang genome (Table 1). Ninety percent of the assembly was in 416 scaffolds and the N50 was 1.55 Mb. Gaps (region composed of at least one N) in scaffold represent 48.3 Mb accounting for 11 % of the assembly.

**Table 1:** Statistics on scaffold assemblies

	V1 (D'hont et al. 2012)	SSPACE	Fusion/joining/splitting/gap re-estimation	IRYS scaffold	GapCloser
Scaffold number	7 513	2 267	1 572	1 532	1 532
Cumulated size	472 210 317	438 736 528	443 852 100	450 994 104	450 697 673
Unknown sites (%)	81 728 542 (17.3)	48 267 272 (11.0)	53 378 493 (12.3)	60 520 497 (13.4)	45 175 659 (10.0)
N50 (scaffold number)	1 311 088 (65)	1 545 585 (52)	2 890 075 (28)	3 014 384 (26)	3 016 874 (26)
N80 (scaffold number)	316 579 (299)	370 770 (242)	491 628 (169)	578 880 (150)	579 793 (150)
N90 (scaffold number)	54 335 (647)	169 980 (416)	201 127 (305)	234 686 (268)	234 825 (267)

### Scaffold correction

**Fig. 2:**
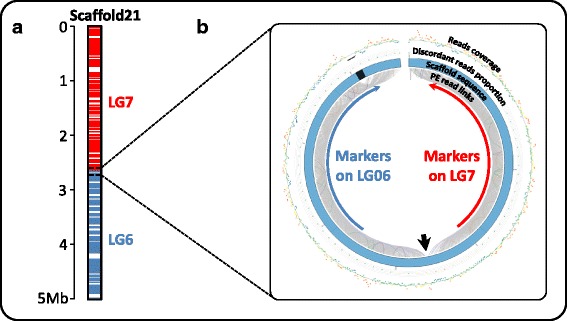
Example of a clue leading to scaffold splitting. **a** Genetic markers mapped onto scaffold21 belong respectively to linkage-group 7 (*red*) and linkage-group 6 (*blue*) suggesting a chimeric misassembly. **b** CIRCOS graphical representation of paired read mapping in the misassembled region. This representation is drawn using *Scaffremodler*’s tools. In the inner circle, links between read pairs are drawn with the following color code: *grey lines* correspond to concordant pairs (correct orientation and insert size), *orange* and *red lines* correspond to discordant pairs with smaller and greater insert size respectively. *Purple lines* correspond to pairs showing reverse-reverse orientation, *green lines*, forward-forward and *blue lines* correspond to pair with complete reverse orientation relative to the paired library construction. The second circle represents scaffold in blue with gaps as black regions. The next circles are scatter plots with warm-cool color code. The first scatter plot presents the proportion of discordant reads on window size of one third of expected read pair insert size. The outer circle represents a scatter plot of read coverage on window size of 100 bases. The *black arrow* points the misassembled region in scaffold21 leading to the assembly of two regions that are not linked

First, we looked for misassembled scaffolds. A total of 33 scaffolds were identified as containing markers from different linkage groups and thus as potentially containing misassembled regions. The misassembled regions were confirmed by the presence of discordant 5 kb LPR in the region. The 36 misassembled regions identified in these 33 scaffolds were then split, resulting in a total of 2,303 scaffolds. Figure 2 shows an example of a misassembled scaffold. Most of the misassembled regions (24/36) resulted from scaffolding errors, potentially due to chimeric paired reads or read misalignment. The remaining misassembled regions (12/36) resulted from contig assembly errors.

**Fig. 3:**
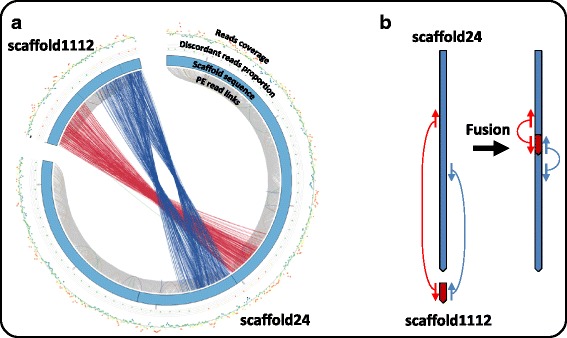
Example of a clue leading to scaffold fusion. **a** Graphical representation of paired read leading to the identification of fusion of scaffold1112 into scaffold24. This representation is drawn using *Scaffremodler*’s tools. In the inner circle, links between read pairs are drawn with the color code described in Fig. 2: *grey* for concordant pairs; red and orange for discordant in size; *purple*, *green* and *blue* for orientation discordant pairs. The second circle represents scaffold in blue with gaps as black regions. The next represents the proportion of discordant reads and the last circle represents read coverage as in Fig. 2. *Red* and *blue* beams linking scaffold1112 and scaffold24 allowed identifying scaffold fusion schematized in (**b**). Inserting scaffold1112 into scaffold24 will correct the discordant red links and correct the orientation of discordant blue links

Second, we looked for potential scaffold fusions and junctions. Based on the analysis of discordant paired-reads from the 5 kb LPR with our semi-automated tools, we could perform a total of 438 scaffold fusions and 293 scaffold junctions, resulting in reduction of scaffold number from 2,303 to 1,572. Figure 3 shows an example of clue leading to scaffold1112 fusion into scaffold24. Additional file 1: Figure S1 shows the mapping of reads on the two borders of scaffold1112 after fusion into scaffold24. Both right and left borders displayed overlapping reads in the correct orientation (Additional file 1: Figure S1, A and B).

At this stage the size of gaps (region composed of Ns) within the new 1,572 scaffolds was re-estimated using the paired reads libraries sequentially resulting in 53 Mb for 12.3 % of the assembly (Table 1). The cumulative size of the new 1,572 scaffolds after gap re-estimation was of 444 Mb. Ninety percent of the assembly was in 305 scaffolds and the N50 was 2.9 Mb.

Finally, BioNano Irys genome map of DH-Pahang was used to order and orient scaffolds into super scaffolds. This step allowed merging of 72 scaffolds into 40 super-scaffolds. A total of 7.1 Mb of gap regions were added during super scaffold construction (Table 1). Finally, 90 % of the assembly was in 268 scaffolds and the N50 was 3.0 Mb with 26 scaffolds. Gaps in scaffolds represented 60.5 Mb for 13.4 % of the assembly.

### Gap closure

Gaps within the 1,532 scaffolds were then tentatively filled with the GapCloser program using the 330 bp pair-end illumina sequencing libraries (50x), generated to correct the first version of the banana *Musa acuminata* reference genome. Of the total of 27,691 gap regions, 9,838 were closed.

### Final assembly

The final assembly (Table 1) consisted of 1,532 scaffolds and showed a cumulative size of 450.7 Mb corresponding to 86 % of the estimated size of the DH-Pahang genome. Ninety percent of the assembly was in 267 scaffolds and the N50 was 3.0 Mb. Gaps in scaffolds represent only 45.2 Mb (10.0 % of the assembly). Twelve of these scaffolds were identified as mitochondrial DNA (cumulative size of 7.2 Mb) using BLAST (blastn, e-value 10^−100^) of mitochondrial coding sequences of *Phoenix dactylifera* (NC_016740). The twelve mitochondrial scaffolds were removed from the final nuclear assembly.

In order to validate the improvements made, the proportion of mapped 5 kb mate pair discordant reads (i.e. wrong insert size and/or orientation) for each scaffold assembly versions was calculated. Over the 82.9 million non-redundant and single mapped pairs, 16.3 million (19.7 %) mapped discordantly on the first version. Over the 82.9 million non-redundant and single mapped pairs 12.3 million (14.8 %) mapped discordantly for the new assembly before gap closure. Over the 80.8 million non-redundant and single mapped pairs 9.6 million (11.9 %) mapped discordantly for the new assembly after gap closure.

### Musa scaffold anchoring

**Fig. 4:**
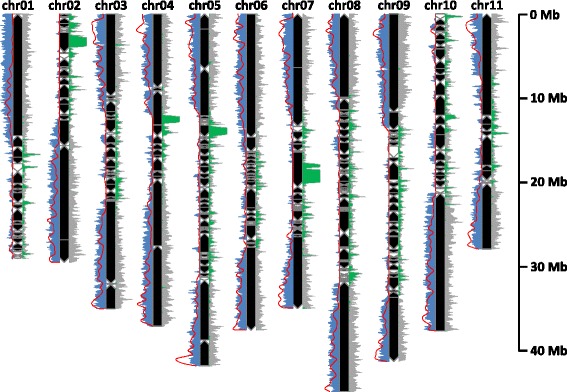
Representation of the new version of eleven pseudo-molecules of *Musa acuminata*. *Black* and *white boxes* correspond to oriented and unoriented scaffolds, respectively. Genetic marker, gene and unknown sequence (‘N’) density are represented in *grey*, *blue* and *green* respectively based on a windows size of 100 kb. The recombination rate (*red curve*) has been calculated on 180 individuals on corrected genetic markers and a sliding window of 500 kb

Genetic markers were then used to assemble scaffolds into pseudo-molecules. Of the 23,430 selected genetic markers, 21,851 that mapped to a unique position were grouped into 11 linkage groups. A total of 248 markers were discarded since they created local discrepancies in scaffolds, clearly attributed to a linkage group based on the majority of the markers. Markers located on small scaffolds for which no linkage group majority could be found were also discarded. The remaining 21,603 markers allowed to order and orient 376 scaffolds into the 11 pseudo-molecules (Fig. 4), with an average of 5.44 markers per 100 kb (Table 2)*.*

**Table 2:** Statistics on marker density on linkage groups

Linkage group	Cumulated scaffold size	Marker number	Marker density (number/100 kb)
chr01	29 067 552	1 384	4.76
chr02	29 509 134	1 502	5.09
chr03	35 017 413	1 920	5.48
chr04	37 104 143	2 489	6.71
chr05	41 848 132	1 924	4.60
chr06	37 589 864	2 234	5.94
chr07	35 025 021	1 744	4.98
chr08	44 883 571	2 728	6.08
chr09	41 302 925	2 136	5.17
chr10	37 671 811	2 023	5.37
chr11	27 952 850	1 519	5.43
Total	396 972 416	21 603	5.44

Finally, a total of 397 Mb of genome sequence was anchored, representing 89.5 % of the nuclear genome assembly (versus 70 % in version 1) and including all scaffolds larger than 1 Mb. Each pseudo-molecule comprised from 16 to 57 scaffolds and N50 in pseudo-molecules varied between 1.4 Mb to 9.9 Mb. The mean N (gap) proportion varied from 5.6 to 12.9 % in pseudo-molecules and was of 25.1 % in unanchored scaffolds (Table 3). Marker linkage in ordered scaffolds can be visualized for each chromosome in Additional file 1: Figure S2.

**Table 3:** Statistics on *Musa acuminata* pseudo-molecule assembly between the first and the new version

	Version 1						Version 2					
Identifier	Scaffold cumulated size	Nb^a^	Scaffold N50	Nb^a^	N in scaffolds	%	Scaffold cumulated size	Nb^a^	Scaffold N50	Nb^a^	N in scaffolds	%
chr01	27 571 529	22	2 245 470	4	3 459 727	12.5	29 067 552	30	1 394 891	2	2 151 480	7.4
chr02	22 052 597	22	1 755 924	3	2 961 122	13.4	29 509 134	27	2 676 329	3	3 555 070	12.0
chr03	30 468 307	22	3 785 391	3	3 981 002	13.1	35 017 413	31	9 733 574	2	2 329 119	6.7
chr04	30 050 316	13	8 856 836	2	3 343 441	11.1	37 104 143	17	7 838 899	3	2 076 824	5.6
chr05	29 375 369	21	2 773 165	4	3 488 635	11.9	41 848 132	52	2 239 696	5	3 976 084	9.5
chr06	34 896 279	30	7 330 853	2	4 472 335	12.8	37 589 864	36	9 841 105	2	2 328 163	6.2
chr07	28 615 304	22	5 244 634	3	4 262 894	14.9	35 025 021	31	6 378 715	3	4 518 654	12.9
chr08	35 437 139	27	2 556 008	3	5 002 970	14.1	44 883 571	57	9 906 416	2	3 821 170	8.5
chr09	34 145 263	37	1 544 587	6	5 397 793	15.8	41 302 925	39	2 119 922	3	3 398 494	8.2
chr10	33 662 572	33	1 266 487	5	5 753 963	17.1	37 671 811	31	1 798 308	3	3 318 350	8.8
chr11	25 512 624	15	7 530 813	2	2 838 651	11.1	27 952 850	16	7 787 879	2	1 979 175	7.1
Mitochondrion	-	-	-	-	-	-	7 218 240	12	616 199	4	37 503	0.5

^a^Scaffold number

**Fig. 5:**
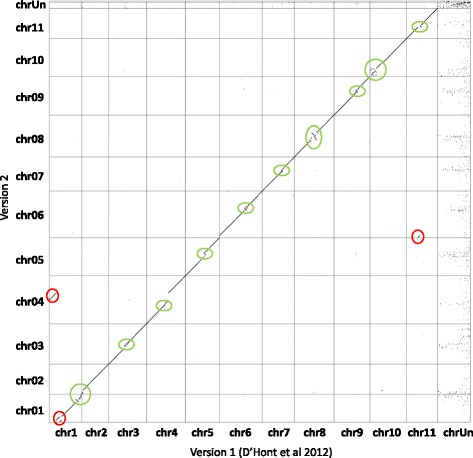
Dot plot comparison of gene order between the initial and the new version of *Musa acuminata* genome sequence assembly. A *dot* represents the position of a gene in the two assembly versions with the initial assembly on x axis and the new one on the y axis. Ruptures in the diagonal indicate differences of gene order. *Red circles* indicate the main differences and *green circles* indicate the variations resulting from the approximate scaffold order in the peri-centromeric regions. For instance, the version 2 of the assembly corrects a significant error between the chromosome 1 and 4

In comparison to the first pseudo-molecule assembly version, we corrected the position of only a few large regions from one pseudo-molecule to another (Fig. 5, Additional file 1: Figure S3). One major change concerned a region that was previously anchored to chromosome 1 and that is now assigned to chromosome 4. These regions of chromosomes 1 and 4 displayed marked segregation distortions that created pseudo-linkages [27] and hampered the anchoring of the first draft assembly that was based on much lower number of genetic markers. Apart from this large change in the assembly, many small modifications were made, representing either anchoring small scaffolds previously unanchored, or small scaffolds reordering. Most of these changes concerned peri-centromeric regions.

### Annotation transfer

Two independent annotations of the initial version of the banana genome assembly were available and both were transferred to the new assembly. The *M. acuminata* transcripts from the first annotation published [27] in addition to several manually curated gene annotation were transferred to the new assembly version. Of the 36,550 predicted genes, 36,154 (98.9 %) genes were transferred to the new assembly version (Table 4). Of the total number of transferred genes, 540 (1.5 %) were located in unanchored scaffolds compared to 2,927 genes (8 %) in the first version. Ninety-six genes were transferred onto the mitochondrial scaffolds. The same transfer was performed for the NCBI Refseq genome annotation. A total of 30,674 (99.9 %) genes of the 30,716 predicted genes were transferred to the new assembly version (Table 4).

**Table 4:** Statistics on annotation transfer between the first release of the assembly and the new release

	First release (D'hont et al. 2012)			New release (version 2)			
Identifier	Pseudo-molecule size (bp)^c^	Number		Pseudo-molecule size (bp)^c^	Number		
		RefSeq^a^	BGH^b^		RefSeq^a^	BGH^b^	Consensus
chr01	27 573 629	2 407	2 836	29 070 452	2 038	2 427	2 372
chr02	22 054 697	1 975	2 328	29 511 734	2 172	2 563	2 517
chr03	30 470 407	2 796	3 251	35 020 413	2 991	3 443	3 371
chr04	30 051 516	2 850	3 368	37 105 743	3 512	4 123	4 018
chr05	29 377 369	2 583	2 972	41 853 232	2 824	3 268	3 215
chr06	34 899 179	3 165	3 700	37 593 364	3 425	4 003	3 896
chr07	28 617 404	2 447	2 764	35 028 021	2 577	2 907	2 918
chr08	35 439 739	2 876	3 458	44 889 171	3 034	3 623	3 489
chr09	34 148 863	2 602	3 110	41 306 725	2 752	3 318	3 157
chr10	33 665 772	2 677	3 157	37 674 811	2 775	3 229	3 155
chr11	25 514 024	2 257	2 679	27 954 350	2 205	2 614	2 521
chrUn_random	141 147 818	2 081	2 927	46 622 217	344	540	543
Mitochondrial	N/A	N/A	N/A	7 218 240	25	96	104
Total	472 960 417	30 716	36 550	450 848 473	30 674	36 154	35 276

^a^NCBI RefSeq genome annotation released the 7 October 2014 and generated with the NCBI Eukaryotic Genome Annotation Pipeline

^b^Banana Genome Hub (BGH) annotation performed by [27], in addition to manually curated genes performed before 08 December 2014 available in the Banana Genome Hub

^c^Including ‘N’ separating scaffolds

Based on the analysis of several manually curated genes, the NCBI RefSeq genome annotation proved to be generally of better quality than the first published annotation in particular because the first annotation over predicted introns. In addition, the NCBI RefSeq genome annotation integrated RNAseq data and predicted alternative transcripts. We thus created a consensus annotation that combined all the manually curated genes, the NCBI Refseq annotation and the predicted genes from the first annotation that were missed by the Refseq annotation pipeline. Using JBrowse in the Banana Genome Hub, these three gene annotations can be visualized as separate tracks. Note that since, we did not perform a new annotation but an annotation transfer, gene fragmentation due to contigs miss-junctions still remains in the new annotated assembly version even if the new assembly version corrected such gene fragmentation. Finally, the consensus annotation contains 35,276 predicted genes with 34,629 (98.2 %) located in chromosomes, 543 (1.5 %) located in unanchored scaffolds and 104 (0.3 %) located in identified mitochondrial scaffolds (Table 4). To avoid any confusion, we modified the nomenclature of Locus tags. For example, GSMUA_Achr5t02570_001 in version 1 becomes Ma05_t02680.1 in version 2.

## Discussion

During the course of this work we succeeded in significantly improving the initial *Musa* nuclear draft genome assembly by reducing the scaffold number by 80 % (7,513 vs. 1532), doubling the N50 value (3.0 vs. 1.3 Mb) and increasing the proportion of assembly anchored to the 11 *Musa* chromosomes by 20 % (70 % vs. 89.5 %) that now include 98.2 % of genes. The decrease of discordant 5 kb read-pairs mapping proportion of 40 % between initial and new version of the assembly support the quality of the changes that were made.

The addition of the 5 kb mate-pair illumina library in the scaffolding process decreased scaffold number by 70 % (7,513 to 2,267) and raised N50 from 1.3 Mb to 1.5 Mb. These results highlighted the importance of medium insert size library during the scaffolding process. Interestingly, the scaffold fusion/junction that we performed decreased further the scaffold number by 30 % (2,267 to 1,572) and significantly impacted the N50 value which nearly doubled. These results highlight the utility and power of the semi-automated tools we have developed. Apart from verifying the newly established scaffolds, the use of BioNano Irys genome maps permitted a few additional scaffold junctions. These maps would have had a bigger impact if they were available earlier during the process [48]. The gap filling step allowed an important reduction of gap regions in the final assembly (17.3 % to 10.0 % between the first and the new assembly versions). The reduction of discordant 5 kb read pairs proportion between the assembly before and after gap filling highlighted the quality of gap closure step performed.

The cumulative size of the new assembly is reduced by 21.5 Mb in comparison with the first genome assembly [27]. This reduction is mainly due to the insertion of small scaffolds into previous gaps of larger scaffolds. The total size of the assembly, lower than expected, can be explained at least in part by difficulties in correctly assembling the repeated fraction of the genome (45S and 5S ribosomal DNA, transposons, retro-transposons and tandem repeats). These repeat-rich sequences are often collapsed into single regions, resulting in a reduced size for the total assembly [5]. For example, 10.6 Mb rDNA have been found in the unassembled reads of DH-Pahang [27].

Saturation of genetic map with DArTseq markers increased the proportion of anchored assembly from 70 to 89.5 % and anchored genes from 92 to 98.2 %. For scaffold anchoring, the classical approach is to construct a genetic map and to anchor the scaffold assembly onto this genetic map to construct a pseudo-molecule. Genotyping errors that are frequent in GBS data can lead to marker miss-ordering in genetic map and to conflict between markers order in genetic map and in scaffolds, when performing the scaffold anchoring. To avoid the tedious step of conciliation between genetic map and scaffolds, we developed a method that takes the advantage of markers already ordered into blocks corresponding to scaffolds. In this context, genotyping error impact is lowered as markers are already partially ordered. The newly anchored regions belong essentially to peri-centromeric regions. However because the proportion of repeated sequence is high in these regions, the marker density is lower (Fig. 4) and the recombination rate is generally very low (or even suppressed) [49–52]. Consequently the scaffold order and orientation in these regions remains tentative.

## Conclusion

The significant improvements made on the banana reference genome sequence will have important impact on the quality of future genetic and comparative genomic analysis. The bioinformatics methods and tools described in this work can be useful to improve draft genome assemblies in other plant species. The pipeline comprises independent modules adaptable to various datatypes. It can be used to improve existing assemblies or in combination with existing automated programs during *de novo* assembly. The improved version of the *Musa acuminata* genome assembly is accessible and can be downloaded in the new version of the Banana Genome Hub at http://banana-genome.cirad.fr/ [53]. Tools are available in command line version on GitHub (https://github.com/SouthGreenPlatform). Most of the options (Modules 2, 3, 4 and 7) are also available on the South Green Galaxy platform under *Scaffhunter* and *Scaffremodler* toolboxes (http://galaxy.southgreen.fr/galaxy/).

### Availability of supporting data

Datasets (contigs, scaffold assembly, Pseudo-molecules, makers matrix and raw data of the genome map) are available through the banana genome hub (http://banana-genome.cirad.fr/) and the 5 kb library is deposited on the ENA read archive (ID number: ERP013665).

## Additional file

Additional file 1: Detailed description of tools and processes used to improve the *Musa acuminata* reference sequence and additional figures. (PDF 1604 kb)

## Abbreviations

BAC: bacterial artificial chromosome
GBS: genotyping by sequencing
HMW: high molecular weight
LPR: large insert size paired reads
NGS: Next Generation Sequencing
TE: transposable elements
WGD: whole genome duplication
WGS: whole genome shotgun sequencing
