# Supplementary material for: Improvement of the banana “Musa acuminata” reference sequence using NGS data and semi-automated bioinformatics methods
Source: BMC Genomics. 2016 Mar 16;17:243. doi: 10.1186/s12864-016-2579-4 (PMC4793746; doi:10.1186/s12864-016-2579-4)
Supplement: Additional file 1: — Detailed description of tools and processes used to improve the Musa acuminata reference sequence and additional figures. (PDF 1604 kb) [file 12864_2016_2579_MOESM1_ESM.pdf]

Supplementary information

Improvement of the banana "*Musa acuminata*" reference sequence using NGS data and semi-automated bioinformatics methods

Guillaume Martin[1], Franc-Christophe Baurens[1], Gaëtan Droc[1], Mathieu Rouard[2], Alberto Cenci[2], Andrzej Kilian[3], Alex Hastie[4], Jaroslav Doležel[5], Jean-Marc Aury[6], Adriana Alberti[6], Françoise Carreel[1], Angélique D'Hont[1*]

[1]. CIRAD (Centre de coopération Internationale en Recherche Agronomique pour le Développement), UMR AGAP, F-34398 Montpellier, France

[2]. Bioversity International, Parc Scientifique Agropolis II, 34397 Montpellier Cedex 5, France.

[3]. Diversity Arrays Technology, Yarralumla, Australian Capital Territory 2600, Australia.

[4]. BioNano Genomics, 9640 Towne Centre Drive, San Diego, CA 92121, USA.

[5]. Institute of Experimental Botany, Centre of the Region Hana for Biotechnological and Agricultural Research, Šlechtitelů 31, CZ-78371 Olomouc, Czech Republic

[6]. Commissariat à l'Energie Atomique (CEA), Institut de Genomique (IG), Genoscope, 2 rue Gaston Cremieux, BP5706, 91057 Evry, France.

*. Corresponding author: Angelique D'Hont

UMR AGAP, CIRAD, TA A-108/03, Avenue Agropolis, 34398 Montpellier cedex 5, France

Phone: +33 (0)4 67 61 59 27

Fax: +33 (0)4 67 61 56 05

Email address: angelique.d'hont@cirad.fr

Table of contents

SUPPLEMENTARY METHODS..3
Module 2: Identification and splitting of scaffold/contig misassemblies 3
Module 3: Scaffold fusions/junctions...5
Module 4: Scaffold gap re-estimation 7
Module 7: Scaffold anchoring...8
SUPPLEMENTARY FIGURES 9
Figure S1: Example of scaffold fusion verification...9
Figure S2: Linkage dot-plots between markers ordered along scaffolds in each chromosome. 10
Figure S3: Comparison of scaffold anchoring between the first version and the new version of *Musa acuminata* pseudo-molecules. 11

SUPPLEMENTARY METHODS

This section describes principles, pipelines and tools used to perform the different modules presented in this work and does not describe tools options. For tools options description, please see the readme and tutorial files available with presented tools on GitHub https://github.com/SouthGreenPlatform.

Module 2: Identification and splitting of scaffold/contig misassemblies

This module aims at identifying and splitting misassembled scaffolds. The complete process and tools used are summarized in Figure 1.

Data requirement:

- Genetic markers (SSR, GBS, DArT)
- Multi-fasta file containing markers sequence
- Paired read fastq files
- Multi-fasta file containing scaffolds/contigs sequence

Module 2 can be described as follows: genetic markers are grouped using JoinMap4.1 software (van Ooijen, 2011) and standard grouping parameters; In parallel, marker sequences are aligned to scaffolds using the *locOnRef* tool; At the end of these two steps, scaffolds harboring markers attributed to more than one linkage group are investigated for evidence of misassembly. To search for the evidence of misassembly, mate-pair reads located in scaffolds comprising genetic markers from different linkage groups are plotted to generate visual data of sequencing coverage at a local scale. The principle is to identify all reads located on these scaffold regions, draw pairing information by joining both reads of a pair and detect a region with no pair overlap that may indicate misassembly.

Paired reads are first mapped onto scaffold regions using *1_create_conf* and *2_map* tools. Identical read pairs (duplicates) and reads having multiple hits are filtered out with the *3_filter_single_pair* tool and *4_filter_sam* tool, respectively. Statistics such as median insert size that is used to re-estimate correctly mapped reads are calculated at this step and scaffold coverage and proportion of discordant reads are also calculated with the *5_calc_stat* tool. Read pairs are then parsed according to their orientation and insert size with the *6_parse_discord* tool. Finally, configuration files with data on reads pair links, coverage and discordant proportion in scaffold regions are generated with *conf4circos* tool and circos pictures (Krzywinski et al., 2009) are generated using the *draw_circos* tool.

Zones that are not covered by read-pairs are visually identified and sequence breakpoint coordinates are identified using a coverage file generated by the *5_calc_stat* tool. Once all scaffold splitting zones are identified, scaffolds are split using *convert2X* and *SplitOnX* tools. The final file is a multi-fasta file containing all scaffolds, including the newly split scaffolds. All scaffolds are renamed by decreasing order of length for module 3 processing.

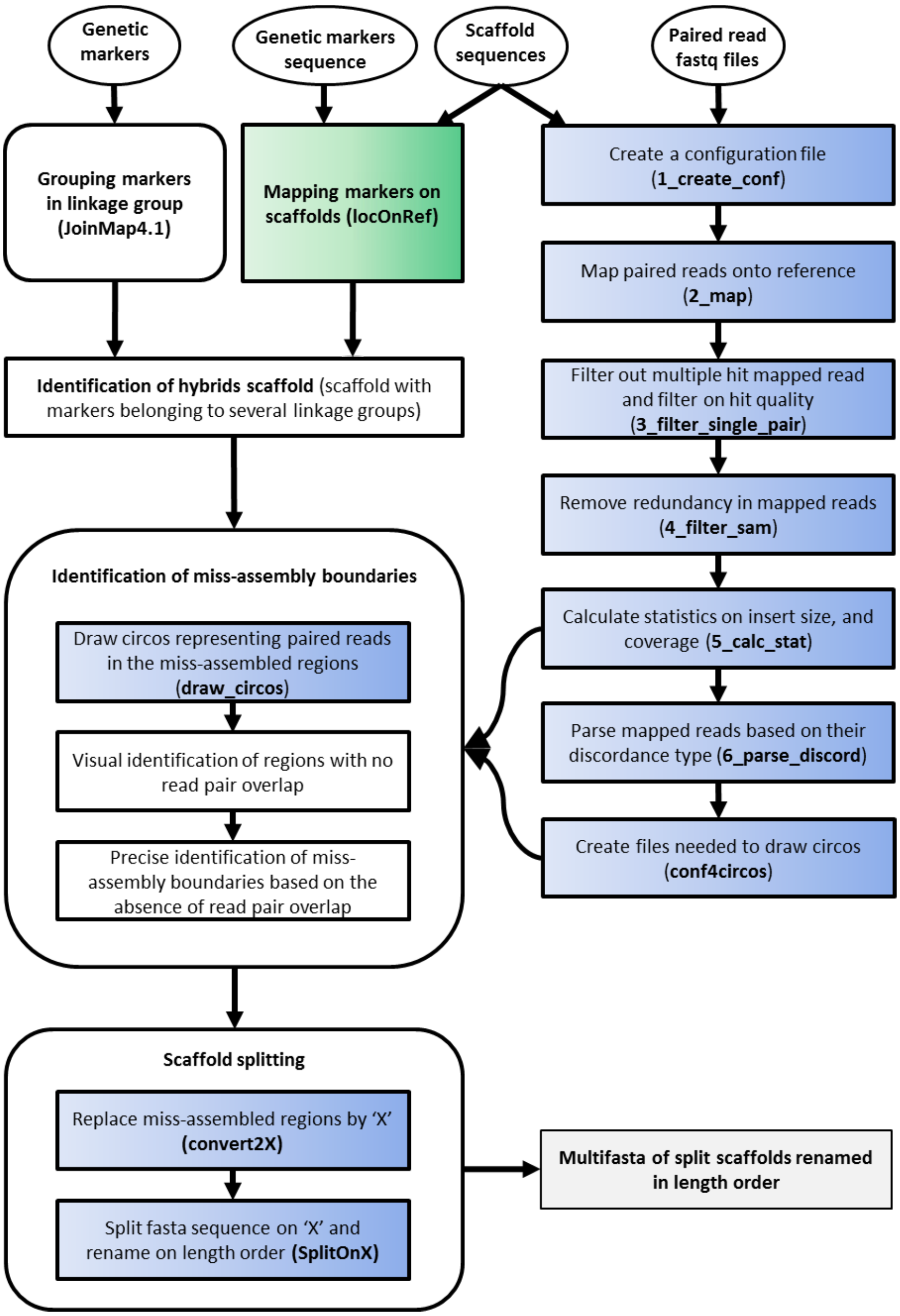

Figure 1: Overview of module 2 that identifies scaffold/contig misassemblies. Tools regrouped under Scaffhunter and Scaffremodler toolboxes are in green and blue rectangles respectively. Grey rectangle presents final output. Program names are in parentheses.

Module 3: Scaffold fusions/junctions

This module identifies scaffolds fusions and junctions missed by automated methods and realizes the fusions and junctions after validation. The complete process and tools used are summarized in Figure 2.

Data requirement:

- Paired read fastq files
- Multi-fasta file containing scaffolds/contigs

To identify scaffold fusions and junctions, reads are mapped onto all scaffolds and filtered out from redundancy (*1_create_conf* to *5_calc_stat* tool). Mapping parameters are adjusted with local statistics (*i.e.* median insert size) as in module 2. Reads are then parsed according to their orientation and insert size with the *6_parse_discord* tool. Discordant zones (*i.e.* zones that include discordant reads in wrong orientation or with incorrect insert sizes) are identified with the *7_select_on_cov* tool. Configuration files with data on discordant reads pairs, discordant proportion in scaffold regions and coverage are generated with *conf4circos* tool (already described in module 2). Tab files containing putative fusion and junction zones and corresponding circos pictures are generated using *look4fusion* tool.

Candidate scaffold fusion zones are manually validated by ensuring that read pairs linking scaffolds are correctly orientated on each circos pictures. Scaffolds are then merged (scaffold fusion) using *fusion_scaff* tool. Once scaffold fusions are performed, fusion zones are verified by running the analysis again from *1_create_conf* to *6_parse_discord* tools on the newly merged scaffolds. Circos configuration files are created using *conf4circos* tool and circos figures representing paired read link at each scaffold fusion boundaries are drawn with *verif_fusion* tool. The presence of correctly orientated read pairs, overlapping the newly assembled fusion zones validates fusions performed.

Candidate scaffold junction zones are manually validated by ensuring that read pair linking scaffolds are correctly orientated on each circos picture. To manage multiple scaffolds joining, scaffolds are first grouped using *group4contig* tool that creates a table ordering scaffolds relative to each other with respective orientations of scaffolds within groups. In some cases the program cannot decide, which scaffold putting one after another (one scaffold extremity linked to more than one scaffold). For these cases, the program takes arbitrarily one scaffold and the other scaffold in reported at the end of the scaffold group (see tutorial). In these cases the table file has been manually re-formatted to order these scaffolds based on precise paired read inspection. When this inspection did not allow concluding, these scaffolds were removed from the junctions table file. This step cannot be automated because, in several cases, some links are missing (especially for small scaffolds and those containing repetitive sequences) and this can lead to ordering errors if automated. Scaffolds are then joined using *contig_scaff* tool.

Scaffold junctions are checked by running again the analysis from *1_create_conf* to *6_parse_discord* tools on newly joined scaffolds. Configuration files for circos are created using *conf4circos* tool and circos pictures representing paired read links at scaffold junction boundaries are drawn with *verif_fusion* tool. Resulting circos figures are inspected for scaffold junction verification by observing correctly orientated read pair overlapping the newly assembled junction zone.

If a scaffold is subjected to a single operation only (junction or fusion), all steps can be performed at once. If multiple events affect the same scaffold (e.g. two scaffolds should be joined and further integrated into a third one, cascade of scaffold fusions), the steps should be performed sequentially by running again the whole process. As an example, if multiple small scaffolds have to be integrated into a single region, these small scaffolds should be either grouped together first (using *contig_scaff* tool) and then integrated as a single scaffold, or sequential fusion steps should be performed using the complete process from *1_creat_conf* to *fusion_scaff* tools until there is no fusion left.

The verification involved mapping and read filtering that are time consuming steps. To save time, these verification steps have been performed on a randomly reduced set of paired-reads representing ¼ of the complete dataset.

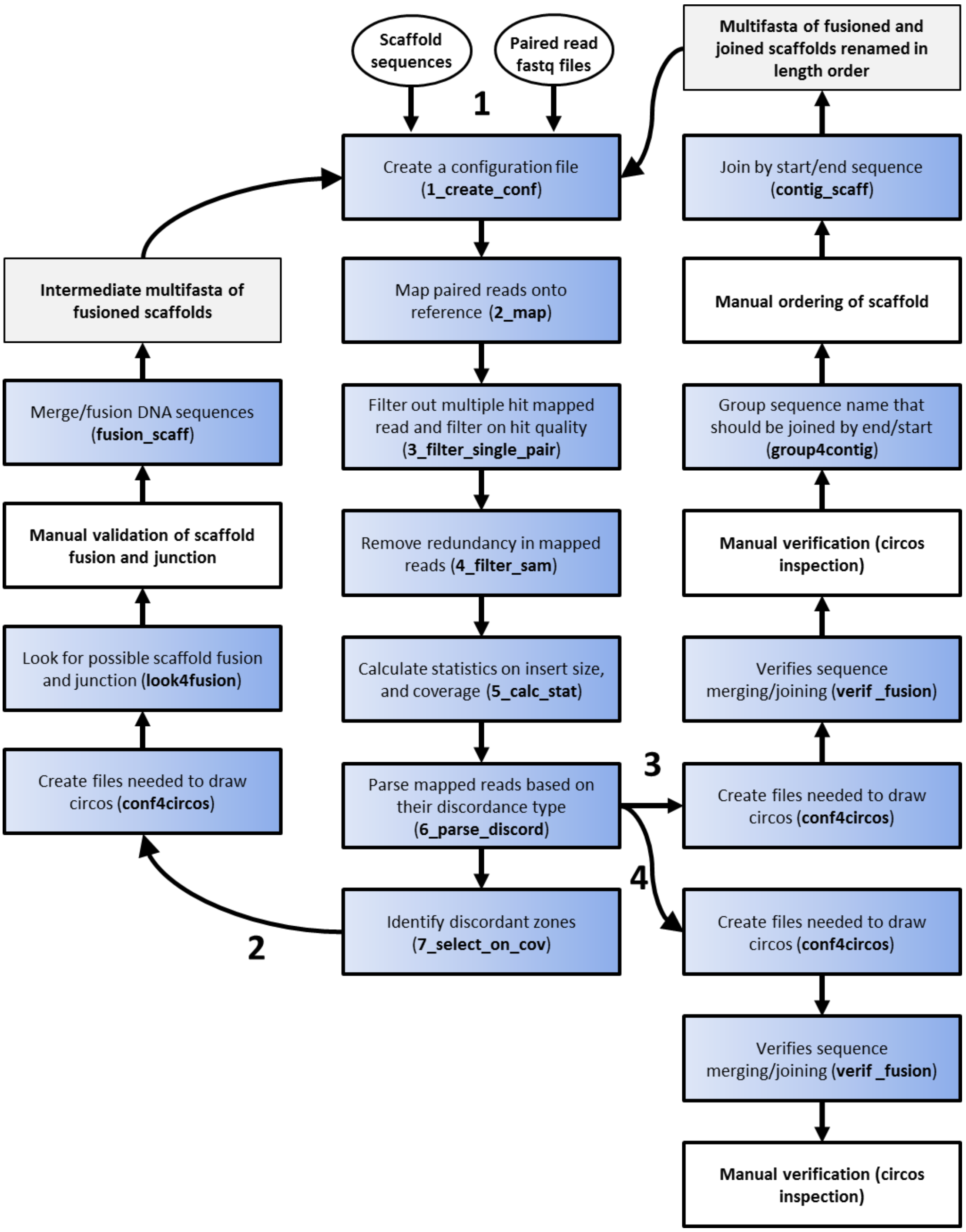

Figure 2: Overview of module 3 that identifies and performs scaffold fusions and junctions. Tools developed are grouped in Scaffremodler toolboxes. Grey rectangles present intermediate and final outputs. Program names are in parentheses.

Module 4: Scaffold gap re-estimation

This module re-estimates scaffold gap size to include the appropriate number of Ns. The complete process and tools used are summarized in Figure 3.

Data requirement:

- Paired reads fastq files
- Multi-fasta file containing scaffolds/contigs

For this module, paired reads are mapped onto the scaffolds (*1_create_conf* and *2_map* tools). Reads having multiple hits and read pair duplicates are filtered out (*3_filter_single_pair* and *4_filter_sam* tools respectively). Gap sizes (i.e. number of N) are then re-estimated with *reEstimateN* tool which uses correctly orientated read pairs in overlapping gap regions. To use this pipeline several times with multiple libraries and to prevent re-estimating already estimated gap regions, the pipeline generates re-estimated gaps with an "E" character for undetermined bases (instead of a classical "N" character). At the end of a gap size re-estimation process, "E" are replaced by "N" in the resulting multi-fasta file.

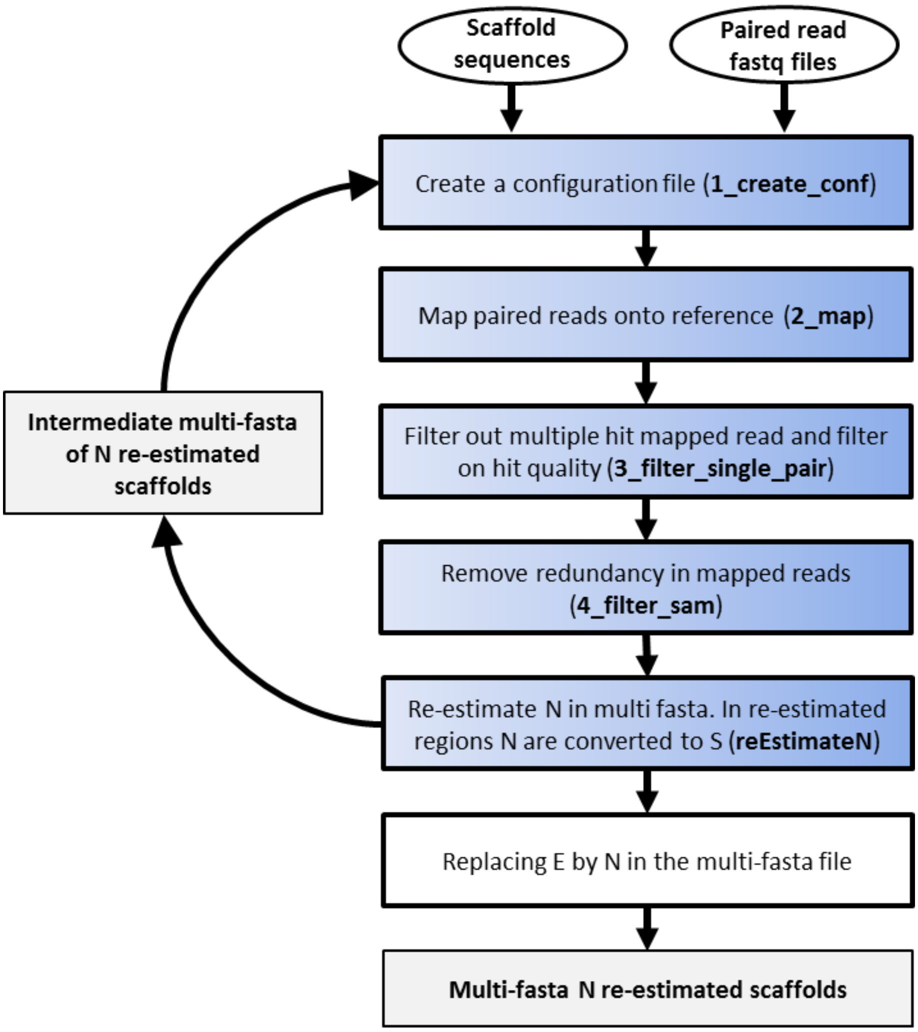

Figure 3: Overview of module 4 that re-estimates gaps in scaffolds. Tools grouped in Scaffremodler toolbox are in blue rectangles. Grey rectangles present intermediate and final outputs. Program names are in parentheses. For details, refer to Material and Methods section.

Module 7: Scaffold anchoring

This step aims at grouping, ordering and orientating scaffolds into pseudo-molecules using GBS markers from a segregating population. The complete process and tools used are summarized in Figure 4.

Data requirement:

- Genetic mapping markers (SSR, GBS, SNP)
- Multi-fasta file containing markers sequence
- Multi-fasta file containing scaffolds/contigs

Genetic markers are grouped using JoinMap4.1 software (van Ooijen, 2011) and standard grouping parameters. Within each linkage group, all pairwise linkage LODs between markers are calculated using JoinMap4.1. The JoinMap pairwise file is then converted into pairwise matrix (*JMpwd2matrix* tool). In parallel, markers sequences are mapped onto scaffolds (*locOnRef* tool). A first order is then calculated using an UPGMA like approach based on mean pairwise linkage LOD with the *UPGMA* tool. Final scaffold ordering and orientation are optimized by performing scaffold permutations and re-orientations with the *reorderient* tool. Scaffold sequences are then assembled into pseudo-molecule (*scaff2chrom* tool). In addition to a fasta file containing ordered scaffold sequences separated by 100 N, an AGP file locating scaffolds into pseudo-molecules is also generated. A dot-plot showing marker linkage along pseudo-molecules is performed and inspected for scaffold miss-ordering (*matrix2ortho* tool).

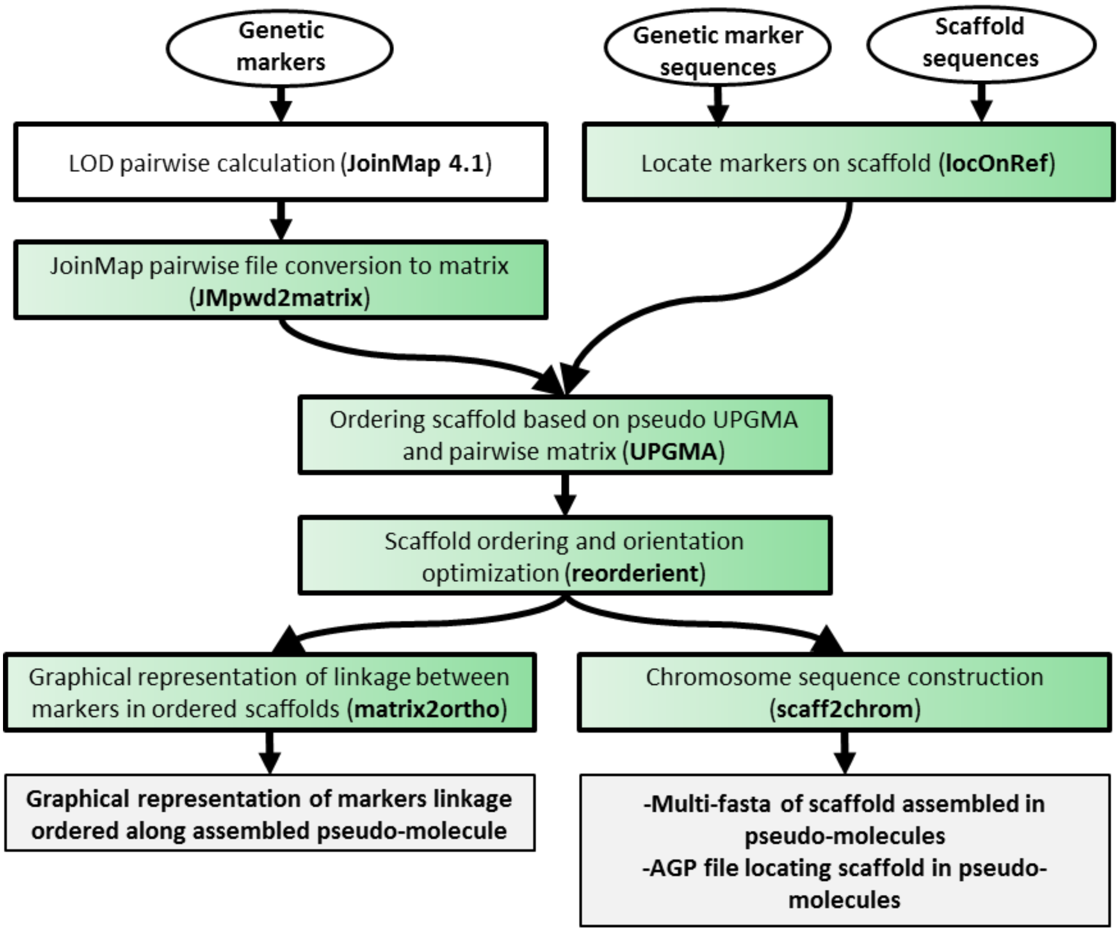

Figure 4: Overview of module 7 that orders scaffolds into pseudo-molecules. Tools grouped in Scaffhunter toolbox are in green rectangles. Final outputs are in grey rectangles. Tool names are in parentheses. For details, refer to Material and Methods section.

SUPPLEMENTARY FIGURES

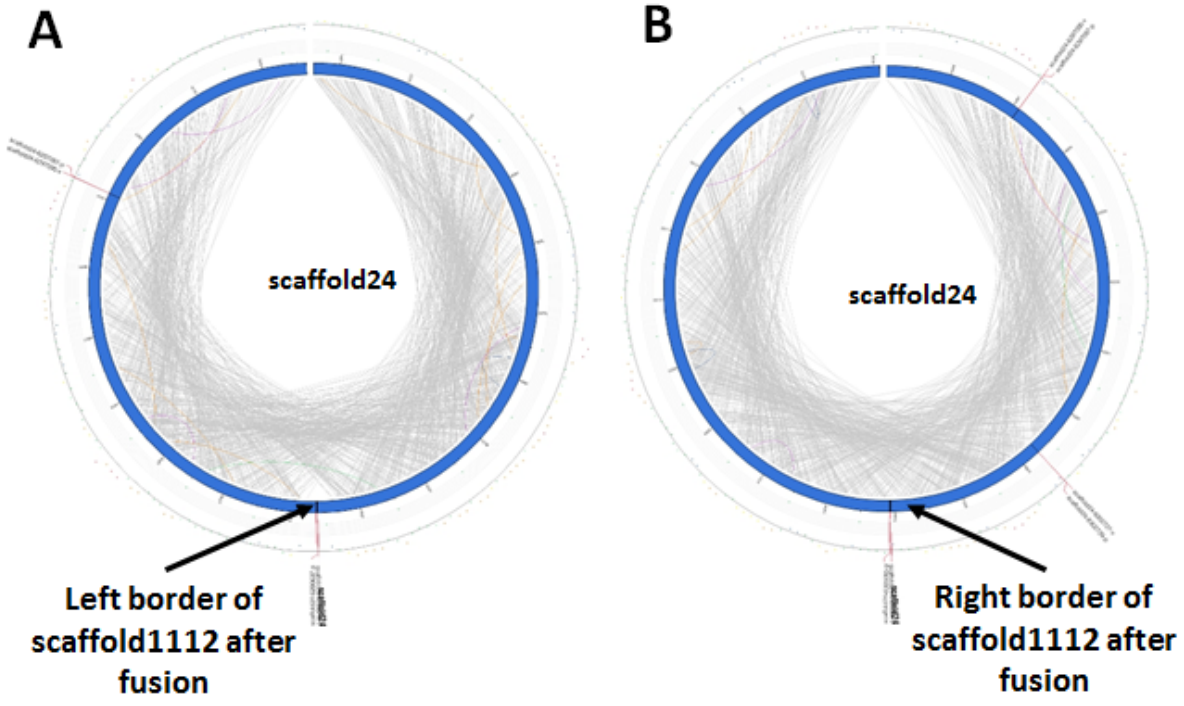

Figure S1: Example of scaffold fusion verification.

Graphical representation of paired read mapping at the boundaries of the fusion of scaffold1112 into scaffold24. (A) Verification of the 5' fusion extremity. (B) Verification of the 3' fusion extremity. This representation is drawn using Scaffremodler's tools. In the inner circle, read pair link are color coded according to their orientation and insert size: concordant pairs (correct orientation and insert size) are drawn in grey, discordant pair due to insert size are drawn in orange and red respectively for smaller and greater insert size. Pair showing the reverse-reverse orientation are drawn in purple, forward-forward orientation in green and pair having the complete reverse orientation relative to the expected (reverse-forward or forward-reverse, depending of paired library construction) are drawn in blue. The second circle represents the scaffolds and black regions locate gaps in the scaffold. The following circle is a scatter plot presenting the proportion of discordant on window size of 1 kb. The last circle represent is a scatter plot of read coverage on window size of 100 bases. The absence of discordant reads overlapping both 5' and 3' fusion zones validate the fusion performed. The presence of well orientated read pairs (grey link) overlapping scaffold fusion regions (left and right borders) confirm scaffold improvement performed.

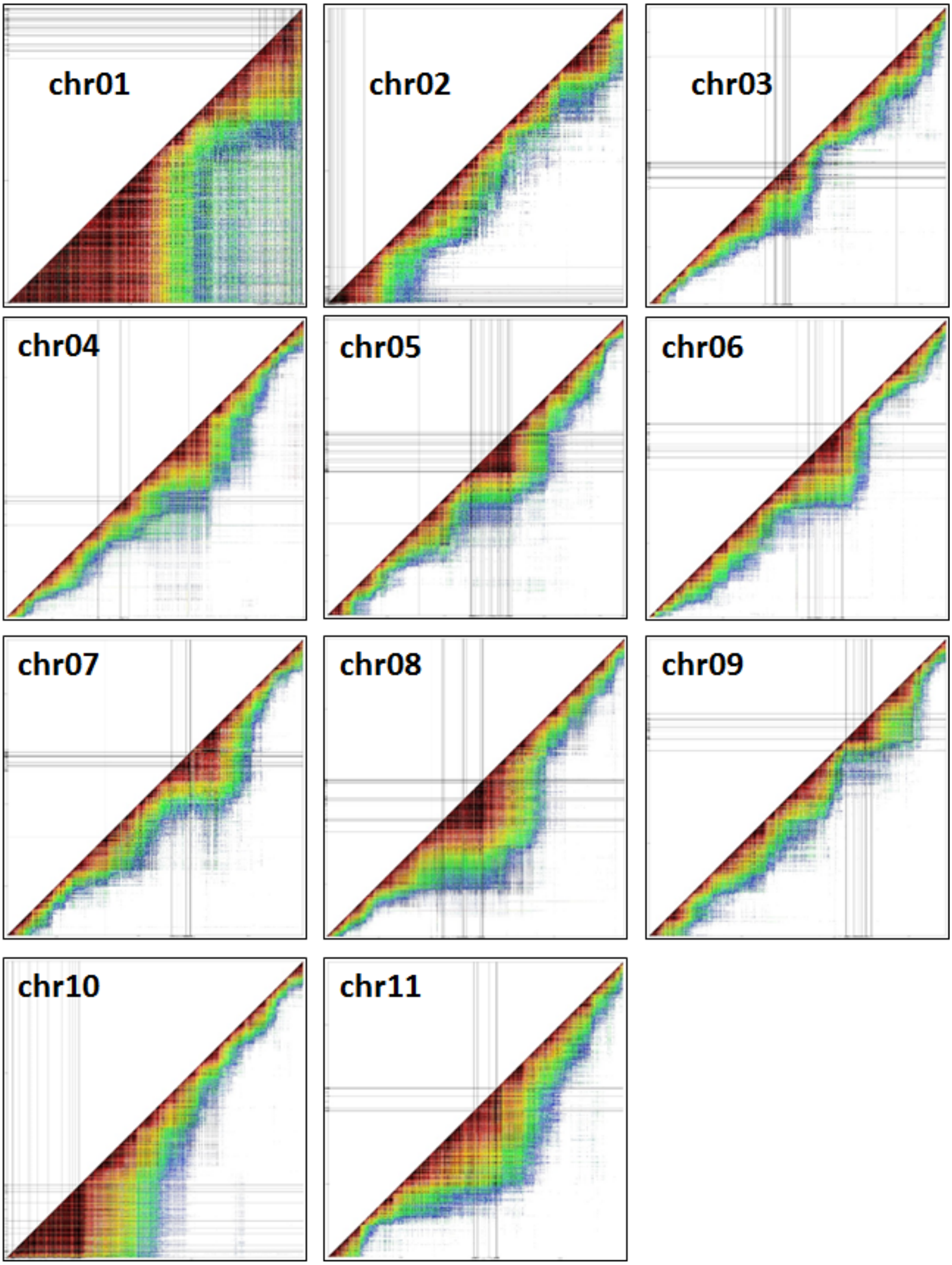

Figure S2: Linkage dot-plots between markers ordered along scaffolds in each chromosome. Each dot represents linkage between two markers. The intensity of the linkage is color coded. Warm color indicates strong linkage and cold color indicates week linkage. Grey bars in dot plots indicate markers belonging to a same scaffold. These dot-plots have been drawn using matrix2ortho tool of scaffhunter toolbox.

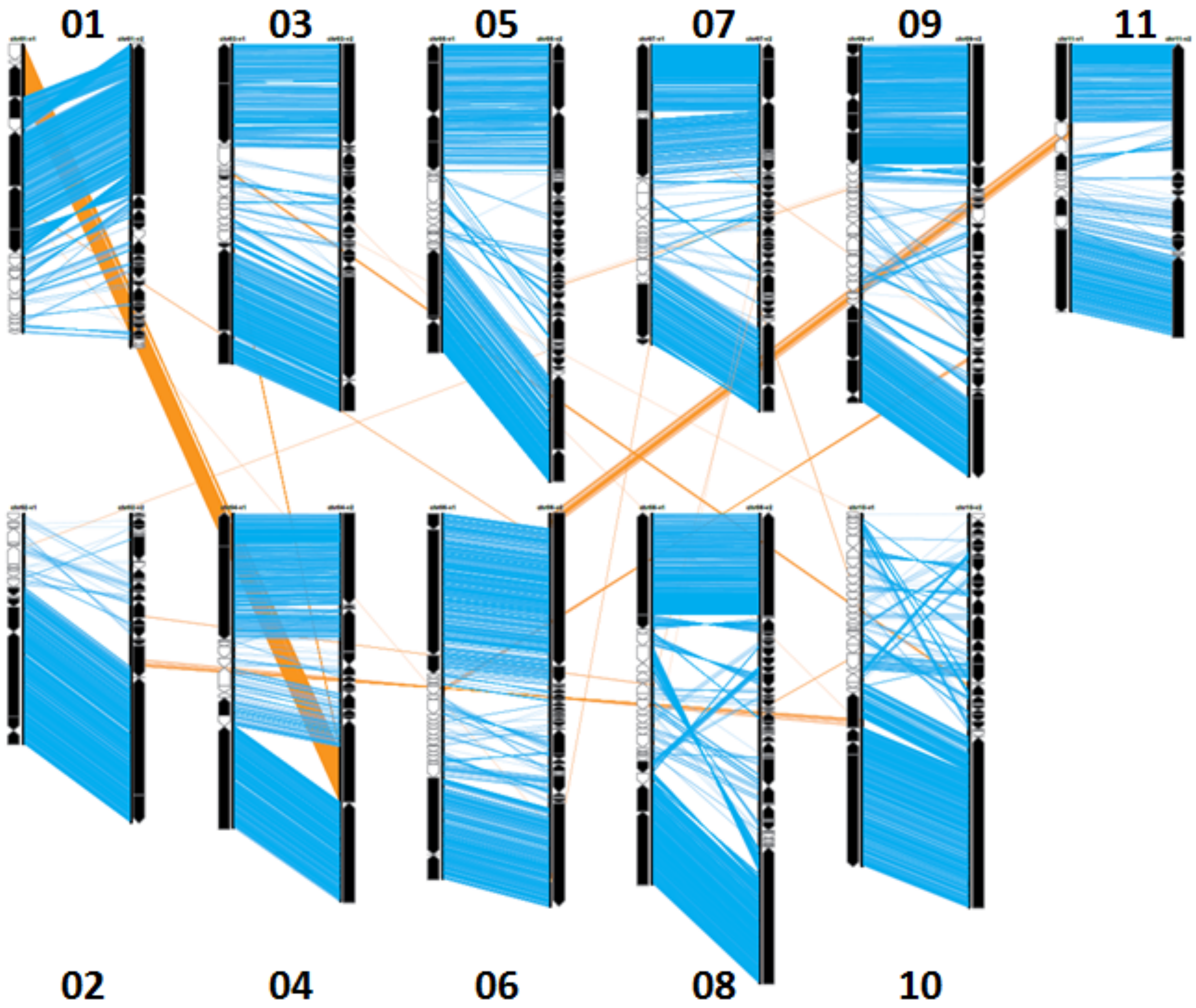

Figure S3: Comparison of scaffold anchoring between the first version and the new version of *Musa acuminata* pseudo-molecules.

For each pseudo-molecule, the new pseudo-molecule assembly (right) is compared to the pseudo-molecule assembly of D'Hont et al (2012) (left). Colored links join identical markers mapped along the two assemblies. Blue: markers located on same pseudo-molecules between the two assemblies. Orange: markers located on different pseudo-molecules between the two assemblies. Boxes symbolize scaffolds and their orientation along the pseudo-molecules.
